# Cyclin-Dependent Kinases (CDK) and Their Role in Diseases Development–Review

**DOI:** 10.3390/ijms22062935

**Published:** 2021-03-13

**Authors:** Paweł Łukasik, Michał Załuski, Izabela Gutowska

**Affiliations:** 1 Department of Medical Chemistry, Pomeranian Medical University in Szczecin, Powstancow Wlkp. 72 Av., 70-111 Szczecin, Poland; pawel_lukasik@yahoo.co.uk; 2 Department of Pharmaceutical Chemistry, Pomeranian Medical University in Szczecin, Powstancow Wlkp. 72 Av., 70-111 Szczecin, Poland; michal.zaluski@pum.edu.pl

**Keywords:** cyclin-dependent kinase, cancer, cell cycle

## Abstract

Cyclin-dependent kinases (CDKs) are involved in many crucial processes, such as cell cycle and transcription, as well as communication, metabolism, and apoptosis. The kinases are organized in a pathway to ensure that, during cell division, each cell accurately replicates its DNA, and ensure its segregation equally between the two daughter cells. Deregulation of any of the stages of the cell cycle or transcription leads to apoptosis but, if uncorrected, can result in a series of diseases, such as cancer, neurodegenerative diseases (Alzheimer’s or Parkinson’s disease), and stroke. This review presents the current state of knowledge about the characteristics of cyclin-dependent kinases as potential pharmacological targets.

## 1. Introduction

Hartwell L.H., in his work on cell division in the baker’s yeast *Saccharomyces cerevisiae,* discovered a great number of genes that control the cell cycle. One of these genes called “START” (later known as CDK1) played a crucial role in regulating the first step of each cell cycle. Next, Nurse P. M. identified and characterized cyclin-dependent kinases (CDKs) and showed that CDK drives the cell cycle by phosphorylating other proteins. In turn, Hunt R. T. discovered the presence of cyclin molecules during the cell cycle, the proteins regulating CDKs [1].

## 2. Cyclin-Dependent Kinases (CDKs)

There are 20 members of CDK family known to this day regulating the cell cycle, transcription and splicing. The kinases are organized in a pathway to ensure that, during cell division, each cell accurately replicates its DNA, and ensures its segregation equally between the two daughter cells [2]. Deregulation of any of the stages of the cell cycle or transcription lead to apoptosis, but if uncorrected, can result in a series of diseases, such as cancer, neurodegenerative diseases (Alzheimer’s or Parkinson’s disease), and stroke [3,4,5]. They also play a key role in the spread of some viral infections, including HIV [6].

The CDK activity is regulated by their association with partner subunits known as cyclins, and without their corresponding cyclin subunit, the enzyme is 40,000 fold less active than in the non-covalent dimer complex; thus, it is essential for functional response [7,8,9,10,11]. Twenty nine cyclins sharing the cyclin box belong to group of proteins that are present in cells during the cell proliferation [12]. Their name derives from the fact that their concentration varies cyclically during the cell cycle; their synthesis and degradation depends on the different stages of the mitotic cell division cycle [13].

Cyclins form a dimer complex with corresponding cyclin-dependent kinases, by interacting with a highly conserved region of 16 amino acid residues, named PSTAIRE motif [14], facilitating large conformational rearrangement of the positions of residues that bind to the ATP phosphate groups. Upon binding to cyclin, the small L12 helix situated at the primary sequence of the T-loop, is altered to become a beta strand, leading to reorientation of the active site and T-loop [15]. Immediately after dissociation of the cyclin-CDK complex, the enzymatic activity of CDK is dramatically reduced, probably due to an alteration in an enzyme’s structure that blocks the active site from any interaction with its metabolites and because of the low concentration of the cyclin [16]. The proteins are inactivated by ubiquitin-mediated proteolysis once they have fulfilled their task.

CDKs are additionally controlled by a series of kinases and phosphatases other than cyclins. The best example of such positive regulator is CDK activating kinase (CAK) which is known to phosphorylate threonine residues at the CDK active sites. This phenomenon was first identified during a work on *Schizosaccharomyces pombe*, where phosphorylation of Thr-167 residue is essential for activity of cell division cycle 2 (CDC2), homologous to CDK1 in the human genome [17]. On the other hand, phosphorylation of the Thr-14 and Tyr-15 residues in human CDK1 exhibits inhibitory effects until dephosphorylation by the dual specificity phosphatase CDC25, which initiates CDK activity [18]. Negative regulatory proteins, also known as endogenous CDK inhibitors (CKIs), react directly with CDKs by blocking the cell cycle progression and transcription.

### 2.1. Cyclin-Dependent Kinase 1 (CDK1)

Cyclin-dependent kinase 1 (CDK1), formerly known as Cdc2, interacts with cyclin B1 to facilitate the transition from the G2 phase into mitosis [19]. This enzyme is further controlled by checkpoint kinases, such as Wee1-like protein kinase (WEE1) and checkpoint kinase 1 (CHK1), which ensure that incompletely replicated or damaged DNA is not distributed to daughter cells [20]. CDK1/cycB1 activity starts to increase in late G2, and continues through prometaphase until the spindle assembly checkpoint is satisfied and the cell enters the metaphase. This complex is activated through the CDC25-mediated dephosphorylation of inhibitory phosphorylation on Thr14 and Tyr15 [21].

### 2.2. Cyclin-Dependent Kinase 2 (CDK2)

In dividing cells, cyclin-dependent kinase 2 (CDK2) is a major cell cycle component that controls the G1/S and S/G2 transitions. CDK2/CycE must phosphorylate Rb to induce S phase entry (*mouse embryonic fibroblasts–MEFs*) [22]. CDK2 has also been shown to regulate the phosphorylation of several transcription factors, inter alia, Myb-related protein B (B-MYB) (mouse, human cells) [23], Myc proto-oncogene (MYC) (U-937 cells) [24], mothers against DPP homolog 3 (SMAD family member 3) (MEFs; epithelial cell lines) [25], inhibitor of DNA binding 2 (ID2) (normal human diploid fibroblasts–TIG-3) [26], forkhead box proteins O1 (FOXO1) [27], and M1 (FOXM1) (human osteosarcoma U2OS cells) [28] and nuclear factor Y (NF-Y) (human HEK293, EJ, and HCT116 cells) [29], which work together to drive the cell cycle through different transition phases. In addition, CDK2 plays key roles in controlling cell differentiation (primary blasts from the human bone marrow–Leu-1-19) [30], proliferation (yeast) [31], apoptosis (podocytes, human) [32], and adaptive immune response (T cells, mice) [33].

### 2.3. Cyclin-Dependent Kinases 4 and 6 (CDK4/6)

Direct inhibition of the cyclin D-CDK4/6 dimer activity prevents cell cycle progression from the G1 to the S phase of the cell cycle. This tightly controlled restriction point is regulated by CDK4/6 complex, which is further controlled by the regulatory subunits D-type cyclins (D1, D2 and D3) and CDK inhibitor p16^INK4a^ ([34]. Activated CDK4/6 complexes are responsible for the phosphorylation of retinoblastoma gene product (Rb) by functionally inactivating it [22]. Phosphorylation of Rb allows dissociation of the transcription factor E2F from the Rb/E2F complexes [35], thus facilitating the subsequent transcription of E2F target genes, such as those for the E-type cyclins (cyclins E1 and E2). By interacting with CDK2, cyclin E hyperphosphorylates RB, further increasing the activity of the E2F target genes, which are needed for initiation of DNA synthesis and entry into the S phase, thereby allowing the cell to proceed through the cell cycle and divide (C33A cells) [36].

### 2.4. Cyclin-Dependent Kinase 5 (CDK5)

**Figure 1:**
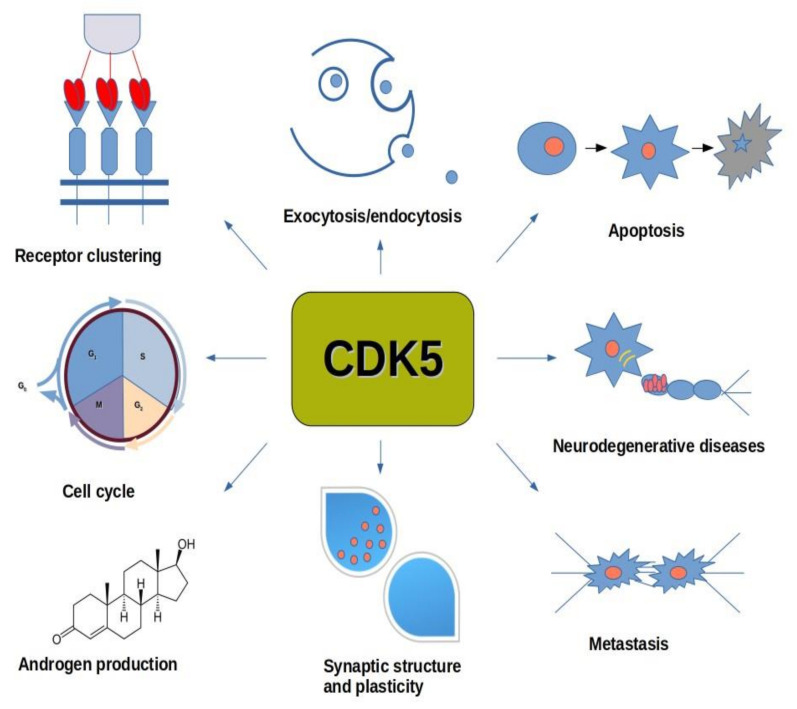
Simplified schematic of the regulation of cyclin-dependent kinase (CDK)5 activity. Involvement of CDK5 in various biological processes.

Despite having high amino acid sequence homology with other CDKs, CDK5 is different as it has been identified to activate various functions in the nervous system, by binding to p35 and p39 neuronal proteins, and their proteolytic cleavage products, p25 and p29, respectively (central nervous system cells) [37]. It has been reported that CDK5 is not activated upon binding with a cyclin and does not require T-loop phosphorylation for activation (bitransgenic 11A or 1A mice). The CDK5 gene is located on chromosome 7q36, and its expression is mediated by Fos and CREB transcription factors in the MEK/ERK signaling pathway, as well as by δFosB (human neuroblastoma SK-N-BE(2)C cells) [38,39]. CDK5 plays a key role in the central nervous system, where it regulates the migration of neurons, the production of neurite connections, as well as their care. CDK5 is also responsible for neuronal migration, synaptic plasticity, neurite growth, as well as maintaining the entire neurogenesis process in adult life (HEK293T cells) [40,41]. In addition to the nervous system, it also plays important roles in cell division, cell differentiation, gene expression, angiogenesis [19,42,43]. Moreover, CDK5 activity reduces secretion of insulin from pancreatic β-cells in response to a rise in the plasma glucose concentration, which was demonstrated using pancreatic β-cells deficient in p35, an activator of CDK5 [44]. The inhibitory phosphorylation by CDK5 on the L-type voltage-dependent Ca^2+^ channel (L-VDCC) at Ser783 prevents the binding of L-VDCC to SNARE proteins, thereby preventing exocytosis of insulin from the cell (pancreatic β-cells) (Figure 1) [45].

Knowing that CDK5 acts as a major factor during embryonic development of the central nervous system and maintains the entire neurogenesis process during adulthood, the aberrant CDK5 activity result in severe disruptions in synaptic homeostasis. Under conditions of environmental stress CDK5 signaling acts as a compensatory mechanism to limit the damage caused by these stressors. However, the irreversible damage leads to cleavage of endogenous p35 to p25 and accumulation of the CDK5/p25 complex. This prolonged activation of CDK5/p25 is associated with neurodegeneration, DNA damage, and cell death [46]. Moreover, studies on p35 knockout mice confirmed a key role of p35 in proper neuronal migration. Mice without p35 survive and are fertile, but display severe cortical lamination defects and are prone to develop fatal seizures [47].

It has been proven that depending on the conditions, CDK5 can either promote or prevent neurite regeneration. CDK5 phosphorylates collapsin response mediator protein 2 (CRMP-2) at Ser-27, thus supporting axonal growth (SH-SY5Y and HEK293T cells) [48,49]. CDK5 may also enable axonal growth by regulating the Axin protein (axis inhibitor). The phosphorylated Axin protein, in turn, stabilizes the microtubular cytoskeleton by inhibiting GSK3β activity (HEK 293T cells) [50]. On the other hand, CDK5 may also interact with the α2-chimerin protein. The complex CDK5-p35 and α2-chimerin enables phosphorylation of CRMP-2 on Ser522, as well as for phosphorylation of CRMP-2 on T514 by GSK3β, which results in inactivation of CRMP-2, destabilization of the microtubular cytoskeleton and inhibition of the growth process [51]. Phosphorylation of p35 on T138 by CDK5 leads to growth inhibition by preventing microtubule polymerization. However, the phosphorylation process on T138 was observed only in utero (HEK 293T cells) [52].

While CDK5 has been well characterized for its functions in the central nervous system, little was known for its role in the cell cycle. Most recent data identify the retinoblastoma protein (Rb) as a crucial CDK5 downstream target. It has been found that CDK5 could regulate the activation state of the tumor suppressor Rb, thereby implicating CDK5 in the regulation of cell cycle progression (MEFs cells) [53].

### 2.5. Cyclin-Dependent Kinase 7 (CDK7)

During the cell cycle CDK7 actively phosphorylates CDK2/cyclin E complex, in order for the cell to cross into the G_1_ state and enter to S phase. On completion of S phase it helps to activate CDK1/cyclin B complex, also called mitosis promoting factor allowing mitotic entry. Formation of a stable dimer of CDK7/cyclin H requires phosphorylation on a conserved threonine (Thr170) in the activation loop of CDK7 and is essential for activity (human cervical tumor cell line–HeLa; human lung small cell carcinoma cell line–H1299; insect cells–SF9) [54,55,56]. Assembly and activity are augmented by a third protein, the RING finger protein MAT1 (ménage-a-trois 1). This trimer, comprising CDK7, cyclin H, and MAT1, forms CDK activating kinase (CAK) a part of the general transcription factor TFIIH (HeLa cells) [57,58,59,60,61]. In this complex CDK7 phosphorylates RNAPII large subunit C-terminal domain (CTD) (eukaryotic cells) [62,63,64,65,66]. TFIIH consists of the holoenzyme IIH, which contains at least six proteins (helicases XPB and XPD necessary for correct transcription initiation and nucleotide excision repair, p62, p55, p44, p34) (yeast) [67]. CDK7 most likely phosphorylates Ser5 in the heptad sequence of RNAP-II. Phosphorylation of the CTD facilitates promoter clearance, initiation of transcription ([68], and recognition by RNA processing enzymes. Levels of CDK7 in cells remain constant and are low [69] and CDK7 is concentrated predominantly in the nucleus (human cells) [70]. Unlike most other CDKs, CDK7 has an additional phosphorylation site within the T-loop (Ser164). It is reported that phosphorylation of this site favors involvement of CDK7 in transcription but is not essential for the regulation of the cell cycle [56,71]. The in vivo activating kinase for human CDK7 phosphorylation is still unknown, but in vitro however active phospho-CDK2/cyclin A can phosphorylate CDK7 [72]. CDK7 also plays an important role in the DNA repair process [73].

### 2.6. Cyclin-Dependent Kinases 8 and 19 (CDK8, CDK19)

CDK8, and its paralog CDK19, together with their regulatory subunits cyclin C, MED12, and MED13 are components of the Mediator complex, which acts as a negative regulator of transcription by directly and indirectly influencing the biochemical activity of RNAP-II and GTFs. CDK8 or CDK19 form a 4-subunit subcomplex with cyclin C, MED12 and MED13 (also known as the kinase-module) that associates in a dynamic fashion with the rest of Mediator complex (HCT116 and HEK293FTcells) [74]. The Mediator phosphorylates the RNAP-II CTD, before RNAP-II is recruited to promoters, causing a disruption in Mediator–RNAP-II interactions as only hypophosphorylated form of RNAP-II can be recruited to promoters (human testis; yeast) [75,76,77]. Moreover, CDK8 dependent phosphorylation of cyclin H at Ser5 and Ser304 residues (a subunit of TFIIH) inhibits TFIIH disrupting transcription initiation (yeast) [78]. Therefore, the interaction of the kinase module with Mediator appears to act as a switch that regulates Mediator–Pol II association [79,80,81]. This would suggest that global gene expression patterns may rely upon precise regulation of the kinase module-Mediator association, but, in fact, this association is regulated by the MED13 subunit [80,81], and knockdown of CDK8 or CDK19 in human cells causes relatively modest effects on gene expression [74,82,83].

Several Mediator subunit aberrations have been linked to the pathogenesis of diverse disorders including cancer (human cells) [84,85,86]. These abnormalities can be either chromosomal [87,88] or gene mutations [89,90,91]. Numerous studies have revealed that CDK8 functions as a key oncogenic driver in several signaling pathways such as Wnt/Catenin signaling (human cells) [91] and TGFβ/SMAD-driven metastases [92]. By now, CDK8 has been found to be implicated in a wide spectrum of cancers, such as breast (human cells) [93], ovarian, and gastric cancer, as well as acute myeloid leukemia [94]. Although CDK19 is less well studied than CDK8, current evidence provided that it is involved in colorectal, breast, and ovarian cancer [95], as well as in fibro- and osteosarcoma (human cells) [96]. Therefore identification and optimization of small molecule inhibitors altering either CDK8 or CDK19 have emerged as promising therapeutic strategies with promising initial results [97,98,99].

### 2.7. Cyclin-Dependent Kinase 9 (CDK9)

The cyclin-dependent protein kinase 9 (CDK9) has been found to regulate the RNAPII transcription elongation. Quantitative Real-time PCR analysis (RT-PCR) of total cellular RNA from cell extracts resulted in identification of two different isoforms of CDK9: 42 kDa protein CDK9_42_ and 55 kDa protein CDK9_55_. The N-terminal regions of both proteins vary greatly in length, with CDK9_42_ consisting of 372 amino acids and CDK9_55_ extended at N terminal domain by 117 amino acids providing additional proline-rich and glycine-rich regions and is expressed from a TATA-box containing promoter. The ratio between CDK9_42_ and CDK9_55_ expression depends on cell type and apparently is governed in a tissue dependent manner. Therefore, the two CDK9 isoproteins have been found to localize to the nucleus (human cells) [100]. CDK9 is activated by interacting with T-type cyclins, T1, T2a, T2b, and closely related cyclin K (yeast) [101]. Like other kinases, CDK9 must be phosphorylated at its activation segment for activity. However it is not phosphorylated by the CAK (CDK7/CycH/MAT1), instead the phosphorylation on Thr186 takes place by autophosphorylation. Phosphorylation on Thr186 is also important for binding of 7SK RNA to CDK9/cyclin T (*Escherichia coli* cells) [102]. CDK9 and its cyclin T partners form the core of positive transcription elongation factor b (P-TEF-b) [103]. The activity of P-TEFb has shown its dependence on its negative regulatory factors, like the small nuclear RNA 7SK (snRNA) and the hexamethylene bisacetamide-inducible proteins (HEXIM1 or HEXIM2). Within the cell two forms of P-TEFb exist: a smaller kinase-active form, consisting of complex of CDK9 bound to its cyclins T or K and a larger, inactive form in complex with HEXIM and 7SK, which is thought to act as a reservoir for the smaller form [104]. External stimuli, such as stress inducing or hypertrophic signals lead to the dissociation of P-TEFb releasing it from the inhibitory complex [105]. Recent studies show that besides the 7SK-HEXIM1–P-TEFb complex, another complex in which a major fraction of nuclear P-TEFb resides is the BRD4–P-TEFb complex (HeLaS3 cells) [106,107]. The BRD4-bound P–TEFb is transcriptionally active and recruited to transcriptional templates possibly due to the ability of BRD4, a co-activator bromodomain protein 4, to bind acetylated histones and the mediator (HeLa cells) [107]. BRD4 preferentially recognizes specific patterns of acetyl histone 3 (H3) and histone 4 (H4) (NIH3T3 cells) [108]. This interaction is significant in stimulating P-TEFb for transcriptional activity through phosphorylation of RNAP-II. Other important transcription factors, such as nuclear factor kappa B (NF-κB), myogenic regulatory factor (MyoD) are recruited to the transcription initiation complex.

P-TEFb governs the RNA transcription elongation by phosphorylation at Ser-2 of CTD RNAP-II [6]. Formation of productive transcription complexes after promoter escape by RNAP-II is also controlled by negative factors. The main negative elongation factor (NELF) consists of four polypeptides. However, NELF needs for activity the two-polypeptide 5,6-dichloro-1-β-D-ribo-benzimidazole-sensitivity inducing factor (DSIF). DSIF/NELF interact with RNAP-II and the RNA transcript respectively making the production of truncated transcripts by polymerase. This promoter-proximal pause stimulates the whole process by providing a checkpoint prior to transcription elongation (*Escherichia coli* cells) [109]. Once P-TEFb phosphorylates the RNAP-II CTD at Ser-2, the Spt5 (p160) subunit of DSIF, and the RD subunit of NELF the repressor pausing is reversed, resuming transcription machinery (HeLa cells) [110,111]. The process of transcription that occurs in almost every living cell at some stage is dependent upon CDK9. The essential knowledge of the functional and structural biology of CDK9 allows to evaluate how and why the suppression of this enzyme can be beneficial in the fight against cancer, AIDS, cardiac hypertrophy, and perhaps even inflammation [6].

### 2.8. Cyclin-Dependent Kinase 10 (CDK10)

CDK10, first mentioned in 1994 [112]⁠, received little interest until it was identified as a critical determinant of tamoxifen resistance to the treatment of breast cancer, combined with endocrine therapy. Additionally, no cyclin partner has been identified up until recently to provide a more detailed analysis of CDK10 functions (MCF7 cells) [113]⁠. Early research on CDK10 had demonstrated that this enzyme exerts a positive control on cell division with its function limited to the G2 or M phase of the cell cycle (HeLa S3 cells) [114]⁠. More recent studies in various human cell lines resulted in: rapid inhibition of proliferation in HeLa cells, G2 phase accumulation in HCT116 colon carcinoma cells or the retinal pigment epithelial (RPE) cell cycle arrest at the G2/M phase, as well a mild, caspase-3/7 induced, decrease in cell viability in MCF7 cells (derived from a ERα-positive breast tumor) [115].

CDK10 kinase activity is regulated by forming a heterodimer with cyclin M, the product of FAM58A, the gene encoding cyclin M [116]⁠. The yeast two-hybrid (Y2H) assay, the most commonly used assay for detecting binary protein-protein interactions, was used to identify whether CDK10 can also regulate transcription. CDK10/cyclin M complex has been found to positively control the N-terminus of the ETS2 transcription factor degradation by phosphorylating it [117]⁠. In addition, CDK10/cyclin M was demonstrated to phosphorylate the protein kinase N2 (PKN2) to repress assembly and elongation of primary cilia, as well as it plays a key role in regulation of the actin network organization, which affects cilia growth (hTERT-RPE1 cells) [118]. Moreover, one single nucleotide polymorphism (SNP) located in CDK10 was found to be associated with familial short stature (FSS) in Han Chinese in Taiwan, which was confirmed in a genome-wide association studies (GWAS) [119].

### 2.9. Cyclin-Dependent Kinase 11 (CDK11)

Recently CDK11, formerly known as PITSLRE, in association with cyclin L has been found to participate in regulating RNA processing and transcription [12]. There are three protein isoforms of CDK11: CDK11^p110^, CDK11^p58^, and CDK11^p46^. The CDK11^p110^ plays an important role in the regulation of transcriptional activity as in vitro studies revealed that p110 interacts with hypo- and hyperphosphorylated forms of RNAP-II and with general transcription factors TFIIF and TFIIS, suggesting that reduction in its activity can block transcriptional activity, whereas readdition of CDK11^p110^ re-established transcriptional activity to some extent. Cdk11^p110^ is also known to modulate RNA splicing and neuronal signaling. The CDK11^p58^ protein isoform is involved in the regulation of mitosis and Cdk11^p46^ is linked to the initiation of apoptosis (CEM-C7 cells) [120,121,122]. Once the transcriptional cycle ends, with mature mRNA strands transcribed, dephosphorylation of the hyperphosphorylated RNAP-II takes place. This process is mediated by a specific TFIIF-associated CTD protein phosphatase, FCP1. After transcription termination, RNAP-II is recycled to carry out another round of transcription (*Escherichia coli;* recombinant human FCP1) [123,124,125].

### 2.10. Cyclin-Dependent Kinases 12 and 13 (CDK12, CDK13)

Initial studies showed that CDK12, in complex with cyclin K, is a transcriptional CDK which mediate a critical step in transition from transcriptional initiation to elongation by phosphorylating RNA polymerase II at Ser2 (HEK293A and HeLa cells) [126,127]. Later analysis found that CDK12 is characterized by the specific upregulation of genes in response to DNA damage, oxidative stress, and heat shock, as well as regulating mRNA splicing, alternative splicing (differential splicing), 3′ end processing, pre-replication complex assembly, and genomic stability during embryonic development. CDK12 is ubiquitously expressed in a number of human tissues and was mainly localized to the nucleus. CDK12 has been implicated in cancer pathology, such as cell invasion, suggesting that aberrant CDK12 expression may have oncogenic properties. Thus, this kinase may become a particularly interesting therapeutic target in prostate [128], esophageal [129], gastric [130], breast [131], endometrial, bladder, uterine, and ovarian [132], pancreatic [133], non-small cell lung cancer [134], lung adenocarcinoma [135], and follicular lymphoma [136]. An increasing number of studies point to the role of CDK12, in cell function and cancer, as an effective strategy to inhibit tumor growth, and its potential clinical use as a biomarker. Although the biological role of CDK13 is not known, its sequence similarity with CDK12 predicts some degree of overlap between these kinases. The CDK12 gene is located on chromosome 7q36, and its closely related CDK13, which is located on 7p14, have been reported to share extensive sequence similarity of 43% with a largely conserved kinase domain (KD) (human cells) [137].

Until recently, CDK9 was considered to be the only elongation-associated Ser2 kinase in metazoans [138]. However, experimental studies from the last 10 years have demonstrated that the CDK12/cyclin K complex also promotes phosphorylation of the CTD of RNA polymerase II at Ser2 in vitro, and depletion of CDK12 resulted in a dramatic reduction in Ser2 phosphorylation in human cells, as opposed to CDK13 depletion, which did not produce any observable change in the levels of phosphorylated Ser2 (yeast) [139]. Depletion of the CDK12/CycK complex results in the downregulation of only a small subset of genes (predominantly long and complex ones) and does not affect global transcription rates [140]. In vitro, knockdown of CDK12 had sensitized cells to DNA-damaging agents. Therefore, these data suggest the involvement of CDK12/CycK in response to DNA damage repair and DNA damage, stress, and heat shock as a master regulator.

Arginine/serine (RS)-rich domains of CDK12 were found to be the critical components of proteins involved in nuclear pre-mRNA processing. The remaining splicing factors are supposedly stored in subnuclear structures known as nuclear speckles (HeLa cells) [141]. Nuclear pre-mRNA splicing is catalyzed by the spliceosome, a large and dynamic complex, which consists of several accessory proteins and five small nuclear ribonucleoproteins (snRNPs) [142]. Further analysis indicated that CDK12 directly phosphorylates pre-mRNA processing factors (human neuroblastoma (NB) cells: Kelly, IMR-32, IMR-5, LAN-1, LAN-5, NGP, SK-N-AS, SH-SY5Y, CHLA-20, CHLA-15, and SK-N-FI) [143]. Nearly all multi-exon human genes are alternatively spliced (human cells) [144], thus aberrant expression may cause splicing defects, which can lead to almost 15% of all genetic diseases (human cells) [145]. In addition, CDK12 was found to indirectly regulate RNA processing by regulating carboxy terminal domain (CTD) of RNA Pol II at Ser2, which governs transcription and mRNA 3′ end processing by interacting with polyadenylation and termination machinery at the 3′ ends of mRNA (SK-BR-3 and MDA-MB-231 cells) [146]. Furthermore, phosphorylation of cyclin E1 at Ser366 mediated by the CDK12/cyclin K complex is necessary for mammalian cell proliferation, which blocks interaction with its binding partner, CDK2, during pre-replicative complex (pre-RC) assembly restricting these events to early G1 phase when CDK activity is low (HCT116 cells) [147].

## 3. Transcription CDKs

The main function of transcription is to allow cells to copy their genomic DNA into messenger RNA (mRNA), which subsequently is translated into proteins. This process is catalyzed by three distinct classes of eukaryotic RNA polymerases: I, II, and III, each composed of two large and 12–15 smaller subunits [148]. However RNA polymerase II is the most studied type of RNA polymerase. It catalyzes the transcription of DNA by synthesizing precursors of messenger RNA and most small nuclear RNAs and microRNAs [149]. RNA polymerase I is responsible for transcription of ribosomal RNA [150], whereas RNA polymerase III synthesizes transfer RNAs (tRNAs) and some small nuclear RNAs found in the nucleus and cytosol [151].

**Figure 2:**
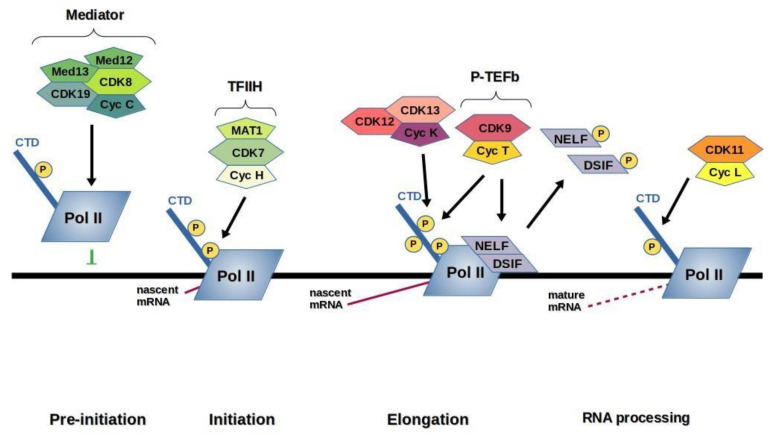
Regulation of transcription initiation and elongation by CDKs. The Mediator phosphorylates RNAP-II. CDK7/cyclin H phosphorylate RNAP-II CTD at Ser 5, which allows promoter clearance by RNAP-II. Phosphorylation of CTD at Ser 2 by CDK9, CDK12, and CDK13 originates in transcriptional elongation. CDK11 regulates the transcriptional activity and the mRNA splicing.

The mRNA transcription is essential for mammalian cell growth and required for the transcriptional initiation, elongation, processing, and termination sequentially. All these aforementioned steps are coordinated by the RNA polymerase II (RNAP-II). The largest subunit RPB-1 of RNAP-II contains at its carboxyl terminus heptapeptide repeat (Tyr1-Ser2-Pro3-Thr4-Ser5-Pro6-Ser7). Each cycle of transcription requires the reversible phosphorylation of this domain. Modifications of CTD phosphorylation by CDKs 7, 8, 9, 11, and phosphatases play a major role in regulation of the transcription [152,153,154]. Recent studies proved that RNAP-II exist in two distinct phosphorylated states: the hypophosphorylated RNAP-IIA and the hyperphosphorylated RNAP-IIO having different roles [155]. RNAP-IIA is normally recruited by promoters during the assembly of a pre-initiation complex (PIC), containing the RNAP-II catalytic core and general transcription factors (GTFs), essentially TFIIB, TFIID, TFIIE, TFIIF, and TFIIH [156,157,158] whereas RNAP-IIo is engaged in the elongation complex [159]. Upon binding of RNAP-II to the promoter the CTD is phosphorylated by CDKs 7, 8, 9, and 11 into the RNAP-IIO (Figure 2), with CDK7 being responsible for transcription initiation. After completion of a nascent transcript, RNAP IIA must be regenerated by CTD phosphatase, an enzyme capable of selectively dephosphorylating the CTD [160]. Moreover, CDK8 is necessary for transcriptional regulation before transcription initiation, CDK7 is involved in transcription initiation, CDK9 being responsible for modulating transcription elongation and CDK11 being involved in regulating RNA processing and splicing. Most recent studies show that CDK12 and CDK13, in complex with cyclin K, mediate the transition from transcriptional initiation to elongation by phosphorylating RNA polymerase II.

## 4. Cell Cycle CDKs

Every cell must replicate all of its material and divide into two daughter cells [7,161]. The division of a eukaryotic cell is composed of four steps: Gap phase-1 (G1), DNA synthesis (S), Gap phase-2 (G2), and mitosis (M). The cell division and mitosis occur during the relatively short M phase. This is followed by G1 phase, the period of cell growth to allow the cell to prepare itself for the DNA synthesis. During the G2 phase, the cell prepares for mitosis. The circle closes with the relatively short M phase, where mitosis and cell division occur. Next, new cells enter the quiescent G_0_ stage, meaning cells lose the ability to further divide for some time. This complex series of events requires appropriate regulation. Thus, there are three main checkpoints that occur during the normal mitotic cycle [161]. Three protein families are required for primary regulation of these restriction points: cyclins, their partner cyclin-dependent kinases (CDKs), and cyclin-dependent kinase inhibitors (CKIs) [162]. The enzymes alter the biological functions of regulatory proteins, so their activity must be regulated by phosphorylation, the presence of activating cyclins and interactions with inhibitory proteins, such as CKIs.

The first checkpoint happens at the end of the cell cycle’s G_1_ phase, just before entry into S phase, when the cell undergoes cell division in place of an alternative developmental process, such as the division delay, or entrance to a G_0_ resting stage. During the G_1_ checkpoint the cell cycle is arrested if environmental conditions make cell division impossible or if the cell passes into G_0_ for an extended period. It is controlled by cyclin-dependent kinase inhibitor p16, a member of INK4 CDK inhibitor family as well as another members of the INK4 family including p15, p18 and p19 by influencing the activity of CDK4/6 [11]. The CKI p16 specifically inhibits the CDK4/6 and ensures that it can no longer interact with cyclin D1 to continue the cell cycle progression. This allows the cell to undergo cell cycle arrest and repair defected DNA. These inhibitors are however expressed continuously throughout the cell cycle, providing a threshold level of suppression necessary to overcome.

Secondly, transition of the G_2_/M boundary, during which any inaccurately duplicated and damaged cells are eliminated. This restriction point is regulated by p53 and is activated in response to DNA damage. Active p53 causes direct interaction of inhibitor proteins, such as p21, with CDK1/cyclin B to arrest passage of cells from G2 into mitosis [11]. Finally, the spindle assembly checkpoint during mitosis that controls precise chromosome alignment and retraction into the two identical daughter cells [11].

**Figure 3:**
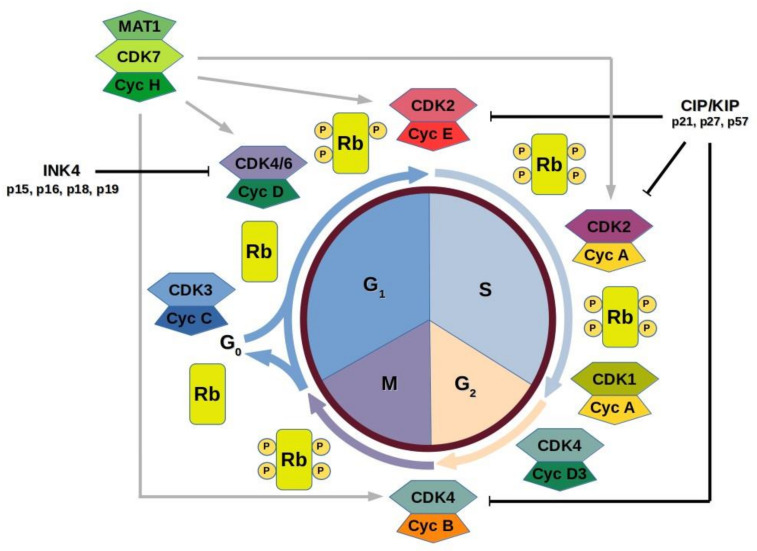
The role of CDKs at different stages of the cell cycle.

CDKs have been found to play a very important role in regulation throughout the cell cycle (Figure 3). CDK3/cyclin C helps the cells to cross the G_0_ resting phase by phosphorylation of pRb [163]. In early G1 phase extracellular signals (e.g., growth factors, mitogenic stimuli) cause the release of D-type cyclins (D1, D2, D3) in association with CDK4 and CDK6 initiate further phosphorylation of the retinoblastoma protein family, including pRb, p107, and p130 [10]. This phosphorylation causes the release of E2F transcription factor, which in turn is able to increase transcription of E2F responsive genes required for cell-cycle progression [164,165]. Among these early E2F responsive genes are those of cyclins A and E [166,167]. In the late G1 phase, CDK2 in association with cyclin E is activated and completes the phosphorylation of pRb, which causes its inactivation. This leads to cross the restriction point at the G_1_/S boundary and to enter S phase. During the S phase, cyclin E is replaced by cyclin A, which by complexing with CDK2 phosphorylate proteins responsible for DNA replication [168,169]. At the cell cycle’s G_2_/M transition cyclin A in association with CDK1 allows the initiation of mitosis [170,171,172,173]. Towards the end of the cycle, cyclin B binds to CDK1 actively participating in and completing mitosis by a series of phosphorylation events [174,175]. Cell cycle exit starts with cyclin D1 transcription cancelled, this causes destruction of the CDK4/cyclin D1 complex. Next, CDK2/cyclin E function is inhibited and on G_1_ cyclin-dependent kinases inhibition, proteins that belong to the retinoblastoma protein family are turned back to their hypophosphorylated active state and cells exit the cycle [176].

All activities and functions of CDK/cyclin complexes are further regulated by two families of CDK inhibitors: the INK4 family (p16, p15, p18, p19), which exclusively bind to and inhibit CDK4 and CDK6, the partner kinases of the D-type cyclins and the Cip/Kip family (p21, p27, p57), which inhibit CDK2/cyclin E, CDK2/cyclin A, CDK1/cyclin A, and CDK1/cyclin B complexes [177,178].

## 5. Cell Cycle and Tumor Development

Any living cell can acquire mutations, especially during its division, which can lead to pathological irregularities. The resultant alterations originate in accumulation of errors in transcriptional regulation and protein expression, influencing the cell cycle machinery. Furthermore, if a mutation affects the error-correcting system within the cell, it causes the uncontrolled production of more abnormal cells, which migrate and disrupt healthy cells finally resulting in tumor [11,178]. From many repressors known to be altered during carcinogenesis, two of them seem to be of great importance, they are retinoblastoma (Rb) and p53 genes.

For each cell to enter S phase, CDKs phosphorylate and inactivate Rb to allow the cell cycle progression. This protein contains 16 sites for potential phosphorylation by cyclin-dependent kinases, such as CDK4/6 and CDK2. Rb remains phosphorylated throughout S, G2, and M phases [166]. p53 protects the genome from any mutagens and if necessary it blocks cell proliferation to prevent abnormalities being inherited. It promotes either DNA repair or cell death through apoptosis [179].

In response to damaged DNA p53 can cause cell cycle arrest. In this case, ataxia-telangiectasia mutated kinase (ATM) phosphorylates p53, its negative regulator mouse double minute 2 homolog, also known as E3 ubiquitin-protein ligase (MDM2), as well as, checkpoint kinase 2 (CHK2) and murine double minute X (MDMX, MDM4), leading to activation of the G1/S checkpoint. The transcriptionally active form of p53 triggers the expression of p21, which binds to and inhibits the Cdk2/cycE complex to prevent downstream protein phosphorylation required for passage into S phase. P53 is also known to control the G2/M checkpoint, by interacting with CDK1/cycB to arrest passage of cells from G2 into mitosis [180]. If the DNA damage is irreversible p53 initiate apoptotic cell death with an involvement of an integral membrane protein B-cell lymphoma-2 (Bcl-2) and Bcl-2-associated X protein (BAX), by inducing mitochondrial caspase dependent apoptosis [181].

## 6. Role of CDKs in Cancer Development

### 6.1. CDK1

Deregulated CDK1 and cyclin B activities were negatively correlated in many cancer types including breast, lung and colorectal tumors [182]. Therefore CDK1 inhibition has been proposed to be an attractive anti-tumor strategy. Indeed, inhibition of CDK1 with a highly selective small molecule CDK1 inhibitor RO-3306 has been shown to be more tumor specific rather than normal cell specific [183,184]. Synergistic inhibition of CDK1 and poly (ADP-ribose) polymerase (PARP), an abundant nuclear enzyme involved in DNA repair, was shown to prolong survival in a spontaneous mouse tumor model without apparent normal tissue toxicity [185]. In addition, the study aimed at determining novel dependencies in GTPase KRas (KRAS–a signal transducer protein, which when mutated enhances tumor cell fitness) mutant cancer cells revealed that the mutant KRAS-driven pancreatic and colon cancer cell models were found to be more sensitive to CDK1 inhibition than KRAS wild-type cell lines in colony formation and cell survival experiments [186].

### 6.2. CDK2

The deregulated expression and activity of CDK2 binding partner cyclins A and E have been associated with a variety of cancer types, including breast, colon, and prostate carcinomas [187,188,189,190]. Overexpression of Cyclin E results in accelerated G1 progression and chromosome instability, which correlates with poor prognosis in patients [191,192]. However, direct inhibition of cyclin E is unlikely as it acts as a regulatory subunit rather than as an enzyme or receptor. Thus, CDK2 as its major catalytic partner has been found to be an attractive pharmacological target. Despite the initial setbacks that CDK2 inhibition by anti-CDK2 shRNA, antisense oligonucleotides, a dominant-negative CDK2, or overexpression of p27^Kip1^ failed to arrest the proliferation of colon carcinoma cells [193]. Moreover, genetic ablation of CDK2 had little effect on cellular proliferation and embryonic development in mice [194]. Later studies on numerous human cancers, susceptible to CDK2 inhibition, provided cause for more optimism in targeting CDK2 as a potentially valuable target. For instance, CDK2 knockout mice remain viable without apparent abnormalities which suggests that CDK2 inhibitors might selectively kill cancer cells without being toxic to normal cells [192]. Additionally, in ovary tumors, elevated CCNE1 level is often correlated with higher CDK2 expression [195]. Deregulation of CDK2 activity is significantly associated with the metastasis of prostate cancer [196], as well as with the development of other cancer types, including breast cancer [197,198], KRAS-mutant lung cancers [199], MYCN-amplified neuroblastoma [200], B-cell lymphomas [201], Glioblastoma multiforme (GBM) [202], hepatocellular carcinoma (HCC) [203], and acute myeloid leukemia (AML) [30]. A clear link between melanocyte lineage transcription factor (MITF) and CDK2 expression levels has been observed in primary melanoma specimens and predict susceptibility to the CDK2 inhibition [204]. Furthermore, the most recent analysis by the number of scientific groups have demonstrated very interesting data for pharmacological CDK2 inhibition through combination strategies. Synergistic interactions of CDK2 and Pl3K inhibitors resulted in apoptosis in glioma and colorectal cancer xenografts [202]. Another combination therapy using CDK2 inhibitors with bromodomain-containing protein 4 (BRD4) inhibitors in MYC amplified medulloblastoma resulted in MYC suppression and promoted apoptosis [205]. The same outcome was observed by combining inhibition of CDK2 and BCL-2 family proteins [206]. The synergistic anti-tumor effect of dual inhibition can also attenuate the development of resistance. Targeting CDK2 overcomes melanoma resistance against Hsp-90 and BRAF inhibitors [207]. Inhibition of CDK2 and CDK4/6 functions has been found to suppress the growth of triple negative breast cancer cells (TNBC), which manifests itself in loss of expression of the RB protein, or high expression of cyclin E, which are thought to confer resistance to treatment with CDK4/6 inhibitors. Moreover, combination of CDK2 inhibition with traditional chemotherapy or radiotherapy, in TNBC, is effective in cases demonstrating high resistance to these forms of treatment [208,209]. CDK2 inhibition has been found to sensitize tamoxifen resistant breast cancer cells both in vitro and in vivo [210].

### 6.3. CDK4/6

Numerous cancers have been found to be particularly sensitive to CDK4/6 inhibition. These genomic or transcriptional aberrations that activate CDK4/6 may lead to alterations in cell cycle machinery genes. For example, amplifications of CDK4 have been manifested in liposarcoma and glioblastoma, and CDK6 have been demonstrated in upper gastrointestinal cancers and neuroendocrine carcinoma of the prostate [211]. These specific genomic translocations and gene mutations can also result in elevated cyclin D levels in tumor cells, for instance, in mantle cell lymphoma. Loss of p16^INK4A^ function has been implicated in many types of cancer such as head and neck, bladder, pancreatic, and lung carcinomas, glioblastoma, as well as malignant peripheral nerve sheath tumors [211,212]. However, the most recent data call into question whether loss of p16^INK4A^ function is actually associated with heightened sensitivity to CDK4/6 inhibition, but rather with upregulated CDK2 activity [213].

Cyclin D-CDK4/6 complexes have also been found to be the key integrators of various mitogenic and antimitogenic signals. For example, many cancers exhibit increased cyclin D levels, which leads to the activation of the RAS–RAF–MEK–ERK pathway, inter alia, by overexpression of the growth factor receptors or by inducing mutations in signaling effector proteins [214]. The hyperactive phosphoinositide-3-kinase/protein kinase B (PI3K/Akt) pathway is also a strong activator of cyclin D1, by preventing its nuclear export and increasing its translation [215]. Moreover, mice lacking CCND1 gene, which encodes the cyclin D1 protein, were unable to develop mammary tumors dependent upon ErbB2 or RAS [175]. However, the clear correlation between elevated levels of cyclin D1 protein in cancer cells and CDK4/6 inhibitor sensitivity is yet to be definitely confirmed. Nevertheless, three newly approved CDK4/6 inhibitors: palbociclib, ribociclib, and abemaciclib have been synthesized. When used in combination with other available therapies for the treatment of patients with hormone receptor positive (HR+), human epidermal growth factor receptor 2 negative (HER2-) breast cancer (HR+/HER2− advanced breast cancer) give hope in search for the cure against this deadly disease [216]. In HR+/HER2− breast cancer cyclin D overexpression is common and loss of pRb function is rare [217].

### 6.4. CDK5

In recent years, more and more evidence has emerged confirming the involvement of CDK5 in the formation and spread of cancers and neurodegenerative diseases, such as Alzheimer’s disease (AD), amyotrophic lateral sclerosis (ALS), frontotemporal dementia (FTD), Huntington’s disease (HD), and Parkinson’s disease (PD), as well as in stroke and diabetes [218,219,220].

Many malignant forms of cancer have been associated with elevated levels of CDK5, such as: medullary thyroid cancer (MTC), hepatocellular carcinoma (HCC) [221,222]. CDK5 phosphorylates the retinoblastoma protein (pRb) thus allowing cell cycle progression by expression of other cyclins and CDKs [222]. The casein kinase 1 (CK1) is phosphorylated by CDK5, consequently CDK5 can indirectly control an array of signaling pathways including cell cycle, apoptosis and DNA repair [223]. When CDK5 phosphorylates CK1, a subsequent decrease in kinase activity is observed [224]. However, the precise mechanisms connecting CDK5-mediated phosphorylation of CK1 on cell cycle, DNA repair, or apoptosis have yet to be discovered.

In the model of mice infected with medullary thyroid carcinoma (MTC), overexpression of p25 led to excessive CDK5 activity and the development of malignant MTC. In contrast, p25-expressing neoplastic mice were able to survive the experiment and it was shown that CDK5 levels in the tumor cells of these mice were significantly lower [225]. In the case of medulloblastoma, disturbances in the expression of CDK5 allow the development of this tumor by deceiving the T lymphocytes, in order to evade detection by the immune system. Lack of expression of CDK5 reduces expression of the transmembrane protein–IFNγ-induced programmed death ligand 1 (PD-L1), which is found to interact with the inhibitory checkpoint molecule PD-1, present in various immune cells. This interaction is important to maintain homeostasis of normal tissues. However, tumor cells can also utilize the same mechanism to evade detection and elimination by T-cells [226]. CDK5 is essential for lymphatic vessel development by phosphorylating Foxc2, the transcription factor which regulates the expression of connexin 37, the junction protein necessary for lymphatic valve formation [227]. Knockdown of endothelial CDK5 results in lymphatic dysfunction and embryonic lethality in mice [228]. In the case of hepatocellular carcinoma (HCC), inhibition of CDK5 expression also inhibits its angiogenesis, which reduces the presence of the hypoxia-inducible factor 1a (HIF-1a), a protein that mediate cellular adaptation to hypoxia, which gives hope for an effective method of curing this type of cancer and other highly vascularized cancers [221]. Recently, it has even been proposed to treat proteins that target CDK5 as possible biomarkers for some cancers. An example would be a lung cancer, where the phosphorylated form of CRMP2 was present in cancer cells, but was not in tumor-surrounding epithelial cells [49].

Numerous studies have demonstrated that CDK5 regulates neuronal migration, layer formation, axon elongation and dendrite arborization during cortical development and adult neurogenesis [229]. In addition, CDK5 activity seems to control a naturally occurring cell motility. However, CDK5 may also regulate cancer metastasis of some forms of cancer, including gastric, prostate, breast, lung, pancreatic, and melanoma [230].

CDK5 may promote cell migration by enhancing pro-migratory Pl3K-Akt Pathway. Recent data show that CDK5 phosphorylates the Gα–interacting vesicle-associated protein (GIV), a protein upregulated in numerous metastatic cancers, which promotes GIV interaction with Gαi, thereby promoting protein kinase B (PKB, Akt) phosphorylation, enzyme important in regulation of metabolism, cell survival, motility, transcription, and cell-cycle progression, actin remodeling, cell migration, and cell survival in podocytes (highly specialized cells of the kidney glomerulus) [230,231].

### 6.5. CDK7

To consider CDK7 as a potential drug target, its dual functions in cell cycle control and gene transcription must be taken into account. Initially it was assumed that inhibition of this enzyme could be prohibitively toxic to normal cells, due to its involvement in gene expression. This assumption should be re-evaluated for two reasons [58]. Firstly, genetic separation of the two CDK7 complex functions is possible by specific mutagenesis, selectively impairing one process [232,233]; or biochemically, by manipulating subunit composition and modification state of the TFIIH-associated CAK [234,235]. The recently published crystal structure of monomeric, inactive CDK7 definitely facilitates the design of such inhibitors [236]. Secondly, transcription by RNA polymerase II depends upon the catalytic activity of CDK7. If CDK7 activity preferentially affects transcripts necessary for transformed dividing cells, targeting this kinase for anti-tumor therapy can be doubly attractive. Inhibition of CDK7 could simultaneously deprive tumor cells of the high CAK activity required for faithful mitoses [237] and limit the synthesis of mRNAs required for other steps in the cell cycle [238], without affecting global transcription in non-dividing cells. It is well known that normal dividing cells have low sensitivity to apoptotic stimuli and exposure to RNAPII inhibition results in growth arrest. Oncogenically transformed cells, on the other hand, are highly sensitive to apoptotic stimuli. This apoptotic sensitivity is counteracted by the induction of one or more survival genes, or apoptotic inhibitors, whose expression depends on sustained RNAPII activity. RNAPII inhibition suppresses the apoptotic inhibitors and leads to apoptosis [239]. However, nuclear receptors, the essential targets for positive regulation by mammalian CDK7, have various functions and important roles in differentiation of numerous tissues. Further careful investigation of interference with these functions and systematic analysis of the role of CDK7 in mammalian gene expression are therefore required. Most recent evidence shows that targeting CDK7 by BS-181 resulted in reduced rates of proliferation, migration, and invasion of gastric cancer cells [239], as well as in combination with BCL-2/BCL-XL inhibitors as a mechanism-based therapeutic strategy could be beneficial in the treatment of Triple-negative breast cancer (TNBC) patients [240].

### 6.6. CDK9

The human body possesses a natural protein p53 that protects cells when exposed to stress caused by oncogenes or DNA damage, by arresting the cell cycle or causing programmed cell death [241]. Mutations of p53 along with pRb are associated with the malignancies in human cells. Knowing that p53 is a transcriptional regulator and a tumor suppressor its absence or deregulation during cell cycle checkpoints results in uncontrolled carcinogenesis. CDK9 is also responsible for transcription of anti-apoptotic factors, that belong to Bcl-2 superfamily: Mcl-1 (Myeloid cell leukaemia-1), Bcl-2 (B-cell CLL/Lymphoma 2), and XIAP (X-linked inhibitor of apoptosis) [242], making CDK9 potential source of inhibition for anti-tumor purposes.

There are two major pathways that can trigger apoptosis. The extrinsic pathway is cell surface dependent, being activated by binding of death ligands to death receptors. The intrinsic, more sensitive pathway occurs in response to cellular stress and is stimulated by the release of mitochondrial cytochrome c [243]. Extrinsic apoptosis can be initiated from outside the cell by activation of a number of pro-apoptotic receptors on cell surface by pro-apoptotic ligands, including Apo2L/TRAIL (receptors DR4, DR5), and CD95L/FasL (receptor CD95/Fas) [244]. Once activated, the death domains of these receptors react with the adaptor protein Fas-associated death domain (FADD) to release the death-induced signaling complex (DISC), and intracellular enzymes, caspases 8 and 10, which later activate caspases 3, 6, and 7 [245], finally converging with the second intrinsic pathway [246].

The intrinsic (mitochondrial) pathway is initiated in response to all sort of cellular stress signals, specifically mitochondrial stress caused by DNA damage or hypoxia and is regulated by p53. This triggers activation of apoptogenic factors, such as cytochrome c in the intermembrane space of the mitochondrion. Once in cytoplasm, cytochrome c binds to apoptotic protease activating factor 1 (Apaf-1), resulting in activation of caspase-9 [247]. Caspase-9, in turn, initiates caspases 3, 6, and 7 to induce apoptosis [248]. This pathway is maintained by a balance between the proapoptotic and antiapoptotic members of Bcl-2 protein superfamily. In the case of carcinogenesis, this balance is corrupted with over-expression of anti-apoptotic factors (Mcl-1, Bcl-2, and XIAP). High levels of Mcl-1 and Bcl-2 have been identified in B-cell chronic lymphocytic leukemia (CLL) [242], a type of cancer that is extremely resistant to many types of treatment. It would then seem valuable to inhibit CDK9 with the aim to suppress the anti-apoptotic transcripts and their proteins, and leading to cell death.

It is well known that normal dividing cells possess low sensitivity to apoptotic stimuli and exposure to RNAPII inhibition results in growth arrest. Because oncogenically transformed cells are highly mutated cells, they are able to escape programmed cell death by the induction of survival genes, or apoptotic inhibitors, including Bcl-2, Mcl-1, IAPs, and surviving [249,250], whose expression depends on sustained RNAPII activity. RNAPII inhibition suppresses the transcription and expression of apoptotic inhibitors resulting in tumor cell death [251]. Normal cells, however, should not be affected by transcriptional inhibition, as majority of normal cells are in quiescent phase and do not require transcription.

### 6.7. CDK11

The larger CDK11^p110^ protein isoform is expressed in many human cancer cell lines such as: osteosarcoma, T-cell leukemia, chronic myelogenous leukemia, and adenocarcinoma [252]. The CDK11^p58^ protein is a mitotic protein kinase, which is specifically expressed only in the G2/M phase of the cell cycle [253], and is closely related to G2/M arrest and apoptosis in a kinase-dependent manner [254,255,256]. CDK11^p58^ is more difficult to detect than CDK11^p110^ and its detection depends primarily on the mitotic characteristics of a particular cell type. Although both isoforms CDK11^p58^ and CDK11^p110^ share the same kinase domain at its C-terminal sequence, the two isoforms possess different functions. Recent studies have shown that CDK11^p58^ is involved in the negative regulation of breast cancer invasion [257,258]. While the larger CDK11^p110^ isoform kinase expression is critical for osteosarcoma and liposarcoma cell growth and proliferation, which have been confirmed via a genome-wide shRNA screening [252,259]. Although, the function of CDK11^p110^ in human breast cancer cell proliferation and growth remains unclear, it has been found that breast tumor tissues and cell lines have high level of expression of CDK11^p110^. In vitro RNAi-mediated knockdown of CDK11^p110^ lead to the inhibition of human breast cancer cell survival and proliferation. Therefore, CDK11p110 plays an important role in the proliferation and growth of human breast cancer cells [260].

### 6.8. CDK12/CDK13

The absence of CDK12 in developing mouse embryos and murine cells, especially in neural progenitor cells (NPCs), usually results in their death during or shortly after birth. These mice exhibit microcephaly and their NPCs accumulate at G2 and M phase, and have lower expression of the cellular DNA damage response (DDR) genes, increased double-strand breaks (DSBs) and increased apoptosis [261]. Moreover, a protein involved in normal cell growth, human epidermal growth factor receptor 2 (HER2), when overexpressed, may promote the growth of cancer cells, including breast, ovarian, bladder, pancreatic, and stomach cancers. High CDK12 expression is significantly correlated with HER2 status, suggesting that aberrant CDK12 expression may have oncogenic properties [262]. Furthermore, carcinogenic role of CDK12 was demonstrated through the expression and alternative last exon (ALE) splicing of genes with long transcripts and large numbers of exons, such as DNAJB6 (DnaJ Heat Shock Protein Family (Hsp40) Member B6), promoted cell invasion and migration in HER2-amplified breast cancer cells [146]. In addition, disruption of CDK12 leads to sensitivity to PARP inhibition, and forced expression of wild-type CDK12 in a CDK12-null cell line model exhibited relative resistance to PARP inhibition [263]. In gastric cancer cells (MKN-7), which display HER2 gene amplification, gene fusions involving CDK12 and HER2 were identified. This data was consistent with the predicted CDK12 protein truncation resulting from the fusion transcripts, but was not in-frame to HER2 [264]. Moreover, enhanced sensitivity to CDK12 has been found to influence tumor-specific (genetic or cellular) expression, such as MYC dependency, EWS/FLI rearrangement and PARP inhibition.

The MYC protein is a transcription factor, which has been demonstrated to regulate a vast number of processes in, both healthy and malignant, cells that impact cell proliferation, growth, metabolism, DNA replication, cell cycle progression, cell adhesion, differentiation, and metastasis [265]. Additionally, it has been identified to influence RNA polymerase II and cell cycle checkpoint control, including GTF2H4, POLR2E, RAD21, and WEE1 [266], as well as induce replicative stress by accelerating the rate of DNA replication, pointing to replication-coupled DDR as a targetable weakness in MYC-driven tumors [267,268]. As MYC governs this many processes, the notion that it could be modulated directly has proven to be difficult [269,270]. However, the overlap between the MYC and cellular functions of CDK12 indicate that CDK12 could be an effective therapeutic target for MYC-dependent cancers. Similarly, suppression of wild-type CDK12 in Ewing sarcoma cells driven by the EWS/FLI fusion oncoprotein, a potent transcriptional activator and transforming gene in this disease, using type VI inhibitor THZ531 (a selective covalent inhibitor of CDK12/13) preferentially decreased expression of DDR genes and was synergistic with PARP inhibitors [271]. Hence, CDK12 loss of function, whether spontaneous or induced, appears to preferentially affect genes that have prominent roles in DNA repair. Some cancer types driven by proto-oncogenes, such as MYC and EWS/FLI, are highly dependent on RNA Pol II transcription [272,273], and the DDRs to maintain genomic integrity during replication [274]. Thus, dual targeting of CDK12 as both a transcriptional coactivator and a DDR regulator could be very beneficial in identifying a promising therapeutic strategy for these cancer types.

CDK12 may have a particularly interesting role as a new therapeutic target in oncology as it has been found to be a clinically relevant biomarker of PARP1/2 inhibitor sensitivity as its inhibition acts synthetically lethal with PARP1 inhibition [275]. Moreover, most of these CDK12 mutations were mutually exclusive with alterations to the breast cancer type 1 and 2 susceptibility proteins (BRCA1 and BRCA2), a tissue-specific tumor suppressor, and a well-recognized DNA repair pathway component. This suggests that primary and acquired resistance to PARP inhibitors could be overcome by CDK12 inhibition in BRCA wild-type and mutated models of triple negative breast cancer [276]. Additionally, CDK12-deficient or BRCA1-deficient cells depend upon the downstream S phase checkpoint kinase CHK1 for survival, and loss of CDK12 or BRCA1 sensitizes cells to CHK1 inhibitors irrespective of p53 status [277].

## 7. Role of CDKs in Rare Developmental Disorders

### 7.1. CDK4/6

Autosomal recessive primary microcephaly (MCPH), also known as Microcephalia vera, is a rare congenital disorder. Patients with MCPH exhibit reduced head circumference and cerebral cortex size, and non-progressive intellectual disability. To date, more than 25 genes have been implicated with MCPH in humans [278]. Following an extensive analysis of databases of genome sequences of consanguineous patients affected by MCPH a single nucleotide mutation was identified in exon 5 of CDK6. This mutation substitutes alanine into threonine at residue 197 within the kinase, which leads to disorganization of microtubules, mitotic spindles and nuclear morphology, resulting in centrosome dysfunction and impaired cell division. In addition, fibroblast cell lines of MCPH patients showed significantly lower growth rate because of a higher rate of spontaneous apoptosis. Although, the biochemical effects of this mutation are yet to be determined, nonetheless first observations have been made. Molecular dynamics simulations revealed that this particular mutation was found in a loop away from the catalytic center, as well as from cyclin and INK binding domains. Interestingly, the patient-derived fibroblasts acted as a loss-of-function mutation because the cellular defects observed were similar in both patient primary fibroblasts and CDK6-knockdown cells. Additionally, the MCPH-causing mutation is limited to a loss of the centrosome-associated functions of CDK6 without affecting its transcriptional activity in hematopoiesis and differentiation [279].

Megalencephaly-polymicrogyria-polydactyly-hydrocephaly (MPPH) syndrome is a rare overgrowth disorder which affect the development of brain architecture associated with intellectual disability and global developmental delay. Few mutations were found in the CCND2 gene encoding for cyclin D2 by whole exome sequencing in many MPPH cases. However, the missense mutation on threonine 280 (Thr280) in the overwhelming majority of patients is most significant because of its involvement in ubiquitin/proteasome-dependent degradation [280,281]. Both glycogen synthase kinase 3b (GSK3b), an enzyme involved in neuronal cell development, energy metabolism and apoptotic pathways, and mitogen-activated protein kinase (MAPK, p38), which is activated in response to oxidative stress, can phosphorylate cyclin D2 on Thr280 to initiate its degradation within the proteasome [282]. The mutated form of CCND2 present in MPPH patients induces a gain-of-function mutation of cyclin D2, meaning it remains stabilized during neural progenitor cell proliferation, which result in uncontrolled cell growth, leading to megalencephaly exhibited in these patients [283].

Since CDK6 loss-of-function mutation causes microcephaly and cyclin D2 gain-of-function mutation results in megalencephaly there should not be a surprise that these two proteins form a heterodimer to obtain an active protein kinase. This unique partnership between CDK6 and cyclin D2 in the regulation of neural cell growth is very rarely seen among other CDK/cyclin pairs governing different processes. Moreover, it has been suggested that cyclins D1 and D2 demonstrate different functions in neurogenesis as they showed differences in expression patterns during forebrain development [283].

### 7.2. CDK5

Recently, a homozygous point mutation of CDK5 has been reported in human lissencephaly (of Latin origin, meaning “smooth brain”) a hereditary brain malformation characterized by the absence or paucity of normal convolutions (folds) present in the cerebral mantle. A group of newborn babies, from a highly consanguineous family in Israel, suffering from a rare and lethal variant of autosomal recessive lissencephaly with cerebellar hypoplasia (LCH) exhibited an agenesis of the corpus callosum, abnormally small heads (microcephaly), as well as severe neurological, dermatological, and facial defects [284]. A pathogenic point mutation was present in intron 8 of CDK5, which was further verified by a whole exome sequencing on one of the affected infants. The same mutation was not detected in more than 200 racially and ethnically matched control individuals. In addition, both patient-derived dermal fibroblasts and brain tissue at mRNA and protein levels showed no detectable levels for the CDK5 protein, in contrast to what was observed in unrelated control. This result is consistent with nonsense-mediated mRNA decay (NMD), a surveillance mechanism that target messenger RNAs (mRNAs) with structural alterations that would otherwise lead to mistakes in protein synthesis, as well as to eliminate any other incorrectly spliced or defective cellular RNAs. Moreover, this mutation causes the complete loss of the CDK5 activity what was confirmed by using a yeast-based complementation assay [285]⁠. Various mice knockout models showed that CDK5 is implicated in normal brain development [286]. Hence, heterozygous silent and intronic mutations in human CDK5 have also been identified in patients with non-syndromic intellectual disability (NS-ID) [287]. However, the detailed mechanism of functional consequences caused by a total CDK5 knockdown resulting in NS-ID have yet to be investigated.

### 7.3. CDK8/CDK19

CDK8, and its paralog CDK19 (>90% sequence similarity between them), together with their regulatory subunit cyclin C form two distinct mediator kinase modules. The Mediator complex is a multisubunit coactivator which allows the assembly of the pre-initiation complex (also called the basal transcriptional machinery) which is involved in the regulated transcription of nearly all RNA polymerase II-dependent genes. Moreover, CDK8 or CDK19 form a four-subunit Mediator kinase modules with MED12, MED13, and cyclin C by reversibly associating with the rest of Mediator complex, which contain enzymatic activity [288]. Although not much is known about the mechanisms by which Mediator kinases exert their functions a several key signaling pathways have been implicated to be governed by these enzymes during development. CDK8 has been demonstrated to regulate Notch, Wnt/β-catenin, and Sonic Hedgehog (Shh) pathways [289]⁠, while CDK19 was found to be highly expressed in a wide range of tissues including fetal eye and fetal brain [290]. Thus far, only one female patient has been identified to be affected by one deep intronic mutation in CDK19. She suffered from microcephaly, multiple café-au-lait spots, congenital retinal folds, hearing loss, and psychomotor retardation. In relation to CDK8, eight heterozygous missense mutations were found by whole-exome sequencing (WES) and whole-genome sequencing (WGS) in twelve unrelated individuals in two different trials. All of these mutations were clustered within the kinase domain, around the ATP binding pocket, without causing major protein instability. This indicates that the CDK8 mutant proteins can still retain some partial ability to bind ATP and cyclin C. Therefore, these numerous phenotypic presentations might stem from small differences in the residual levels of CDK8 activity. The patients exhibited mild to moderate developmental delay, facial dysmorphisms, motor unit hypotonia, as well as emotional and psychological symptoms of behavioral disorders, such as autism spectrum disorder (ASD) and attention deficit hyperactivity disorder (ADHD). Some of these individuals presented congenital heart defects, sensorineural hearing loss, agenesis of the corpus callosum, as well as ocular and anorectal malformations [290]⁠.

### 7.4. CDK10

In the absence of CDK10, endogenous ETS2 protein levels are increased. The overexpression of ETS2 have been implicated in the pathophysiological features of Down syndrome (DS), and alterations in its expression in mice result in spine malformations [291]⁠. On the other hand, CDK10 mutations lead to Al Kaissi syndrome, an autosomal recessive developmental disorder characterized by growth retardation, spine malformation, particularly of the cervical spine. These mutations result in frameshifts or internal truncations which lower CDK10 levels probably through NMD of the mRNAs. Hence, these anomalies are thought to be the loss-of-function CDK10 mutations [113]. Conversely, another study showed that one 11-year old female patient with globally similar symptoms, but also with abnormal primary cilia, which is observed in STAR syndrome patients with cyclin M loss-of-function mutations or in the experimental CDK10/cyclin M knockdown. The homozygous single nucleotide deletion in the 11th of the 13 exons of CDK10 results in shorter, less abundant primary cilia. The mutant CDK10 mRNA does not undergo the NMD pathway. It is assumed that a shorter, 307 residues long CDK10 protein is produced, instead of the wild-type isoform (360 amino acids), which contains 17 missense amino acids at its C-terminal. If expressed, this shorter isoform of CDK10 would be devoid of the C-terminal bipartite nuclear localization sequence, which is usually produced in the wild-type protein system [292]. Therefore, it can potentially retain its interaction with cyclin M, as well as keep some functionality exerted by the wild-type CDK10 [112,115]⁠. Moreover, one single nucleotide polymorphism (SNP) located in CDK10 was found to be associated with familial short stature (FSS) in Han Chinese in Taiwan, which was confirmed in a genome-wide association studies (GWAS) [119]⁠.

Mutations in FAM58A gene cause a severe human developmental disorder, called STAR syndrome, which is characterized by toe syndactyly, telecanthus, and anogenital and renal abnormalities [293]. All patients suffer from growth retardation, and some of them presenting additional malformations such as multiple ocular abnormalities, skeletal defects, tethered spinal cord or lax joints [294,295,296]. It is worth noting that these malformations affecting the FAM58A gene are positioned on the X-chromosome, which is consistent with the fact that only females suffer from this syndrome. All deletions or mutations seem to be very rare (apart from the four mother–daughter pairs reported), and the majority of these additional anomalies are associated with the significant deletions which are broader than just the FAM58A locus [296]. In most cases, these massive deletions prevent cyclin M expression, which leads to the conclusion that the heterodimer of CDK10/cyclin M is impaired in STAR syndrome. This results in higher levels of ETS2 protein [116], which in Ets2 transgenic mice leads to skeletal and cranial abnormalities [297]. Since suppression of either cyclin M or CDK10 promotes ciliogenesis, it can be argued that Star syndrome can be classified as another type of ciliopathy [109].

The distinction between the STAR and the Al Kaissi syndromes is not always clear, as it is difficult to group patients under particular syndrome label, because both cyclin M and/or CDK10 exert more functions than those exerted by the protein kinase heterodimer. Furthermore, an extensive investigation whether these functions can be at least partially compensated by other members of cyclins or CDK kinases is needed to better understand the complex mechanisms underlying these syndromes. One of such examples is co-precipitation of cyclin G2 with either exogenous or endogenous CDK10 [297,298]⁠.

### 7.5. CDK12/CDK13

Like CDK12, CDK13 has also been found to be involved in pre-mRNA splicing regulation because it possesses serine-arginine (SR)-rich region in its N-terminus. CDK13 interacts with cyclin K as its regulatory subunit. The CDK13/cyclin K heterodimer phosphorylates the highly repetitive carboxy terminal domain (CTD) of RNA polymerase II and participates in gene expression control [299]⁠.

The great number of clinical cases described in the studied area demonstrate missense mutations in CDK13 kinase domain, with many variants targeting the highly conserved asparagine residue at position 842. These mutations have been identified as a new source of syndromic intellectual disability in which diagnosed patients exhibit distinctive craniofacial characteristics, feeding problems in infancy, as well as the brain malformations and structural heart defects [300,301,302,303]⁠. Moreover, four unrelated Chinese children affected by neurodevelopmental disorder with facial dysmorphism have been reported to harbor potentially pathogenic CCNK gene (gene responsible for coding of cyclin K) mutations with de novo inheritance. Three of them harboring specific deletions in the 14q32.3 region, which is involved in the expression of 3 different genes other than CCNK and one individual harboring a missense mutation in the CCNK gene (replacement of Lys111 with Glu). All patients presented poor intellectual, motor, language skills, and abnormal dysmorphic facial features. Since all four patients displayed similar phenotypic profiles, a de novo missense variant of CCNK, found in the fourth individual, was chosen for in silico atomic structure analysis. This experiment has demonstrated that the mutated residue was mapped in the heterodimeric interfaces with CDK12 and CDK13. The adjacent amino acid is likely to destabilize both complexes, leading to the inhibition of both kinases. The above data indicate that the most likely pathogenic mechanism, in all four patients, may be a result of haploinsufficiency [304].

The exact functions of CDK13 and cyclin K in development are still to be explored. However, CDK12, CDK13 and cyclin K proteins are highly expressed in murine embryonic stem cells self-renewal [305]⁠, as well as both CDK12 and CDK13, were demonstrated to promote axonal elongation through a common signaling pathway which controls CDK5 expression at the RNA level [306]⁠. Functional assays in zebrafish larvae produced similar dysmorphic features, being reminiscent of those identified in the Chinese children, support a causal role of CCNK variants in neurodevelopment [304]⁠. Additionally, cyclin K was found to function as a CDK9 regulatory subunit, which would suggest that cyclin K interacts with multiple protein kinases, not only with CDK12 and CDK13 [101]⁠.

## 8. Role of CDKs in Other Disorders

### 8.1. CDK5 and Neurological Diseases

Due to the fact that CDK5 plays important roles during the development of the nervous system; therefore, any disruption of CDK5 activation can lead to many neurological diseases. For example, Alzheimer’s disease is characterized by the formation of neurofibrillary tangles, which arise in response to hyperphosphorylation of CRMP-2 responsible for axonal growth, as well as the Tau protein [307,308,309]. Cellular stress also over-activates CDK5 because it leads to the formation of p25, and thus to hyperphosphorylation of the Tau protein, leading to abnormal cell cycle, disruption of synapses (synaptotoxicity), and neuronal loss [310]. The reduction or complete inactivity of CDK5 is also harmful, which can cause neurological diseases or intellectual disorders, such as severe type 1 neurofibromatosis or schizophrenia [311,312] and spontaneous attacks [313].

Amyotrophic lateral sclerosis (ALS) is a fatal disease, which is characterized by the progressive death of upper and lower motor neurons within the brain and the spinal cord, and eventual loss of motor function. The presence of ubiquitinated protein aggregates in affected motor neurons, and their progressive buildup leads to abnormal functioning of muscle tissues. This causes myasthenia, dysphagia, atrophy, and, eventually, loss of control of all muscles responsible for voluntary movements. Abnormal CDK5 activity hyperphosphorylates tau and neurofilament (NF) proteins, leading to microtubule network destabilization, neuronal retraction, and apoptosis [314]. Neurofilament proteins constitute the cytoskeletal elements that form and maintain cell shape and facilitate the transport of particles and organelles within the cytoplasm. Neurofilament proteins have long been assigned a role in the pathogenesis and progression of ALS [315]. CDK5 is considered the most important neurofilament kinase that is involved in other signal transduction pathways, such as the mitogen-activated protein kinase and myelin-associated glycoprotein pathways, which, in turn, influence the phosphorylation of neurofilaments and other cytoskeletal proteins [316].

Mutations in the gene encoding the superoxide dismutase 1 (SOD1), have been first implicated in progressive motor neuron death and paralysis as a cause of familial forms of ALS [317]. Most recent studies point to an involvement of deregulated CDK5 activity in the pathogenesis of mutant SOD1-mediated disease and that the inhibition of this activity may enhance motor neuron survival [318]. Transgenic mice expressing a mutant SOD1 gene display increased ratio of p25/p35, in addition to hyperactivation and aberrant localization of CDK5. CDK5/p25 complex results in hyperphosphorylation and abnormal accumulation of the neurofilament protein, its heavily phosphorylated axonal variant (NF-H), a common feature of ALS. An overexpression of NF-H in mutant SOD1 mice significantly increases their lifespan, which implies that NF-H might act as a competitive substrate for CDK5 [319]. Hence, developing efficacious therapeutic strategies for treatment of ALS must consider the potential of CDK5 inhibition.

### 8.2. CDK9 and HIV

Many viruses exploit host cell forcing it to replicate and transcribe their genomes, including human immunodeficiency virus type 1 (HIV-1). Numerous antiretroviral forms of treatment are being introduced to suppress HIV-1 transcription. However the development of mutations of HIV-1 led to the emergence of multidrug-resistant viruses, urging the need for new anti-HIV treatment strategies [320].

It has been found that transcription of HIV-1 mRNA is facilitated by phosphorylation of RNAP-II CTD by CDK9 [321]. HIV transcription from the long terminal repeat (LTR) is modulated by the combined activity of cellular initiation factors and the virally encoded regulatory protein, the transcriptional transactivator (Tat) [322,323]. This polypeptide of 86–101 amino acids, which is required for efficient virus replication, interacts with P-TEFb resulting in a recruitment to the HIV promoter ipso facto depriving BRD4 its role as a recruitment protein [324]. Tat directs P-TEFb to RNAP-II through cooperative binding to TAR, transactivation response element, a viral RNA stem-loop structure [325]. This trimer, containing Tat, TAR and P-TEFb leads to conformational changes that activate the CDK9 kinase. Tat-activated CDK9 phosphorylate RNAP-II CTD heptapeptide at serines 2 and 5 and the presence of CDK7 is not required [326]. Next, Tat-P-TEFb phosphorylates some specific elongation factors, such as Spt5, a subunit of the DRB sensitivity-inducing factor (DSIF) [327], and the RD subunit of the elongation repressive factor (NELF) [328]. These phosphorylations enhance the processivity of the transcription elongation complex.

The latest research shows that Tat interacts solely with the active P-TEFb complex. Thus the coordinated inhibitory action of HEXIM1/7SK snRNA complex prevents Tat binding. It is thought that Tat overcomes this obstacle by liberating P-TEFb from its negative factors HEXIM1 and 7SK snRNA by hijacking it from these regulators. This allows Tat to antagonize the HEXIM1 interaction with cyclin T1, disrupting the 7SK snRNA of its function to turn the HEXIM1 into a P-TEFb inhibitor leading to a significant increase of the free form of P-TEFb for recruitment for efficient HIV-1 transcription [329,330].

### 8.3. CDK9 and Cardiac Disorders

Cardiac hypertrophy is the result of cardiomyocyte enlargement of the heart muscle (myocardium) as a response to myocardial injury, such as myocardial infarction or prolonged periods of high blood pressure (hypertension). Normally hypertrophy can be seen during embryonic stages of heart development, where CDKs 7 and 9 function as stimulators of hypertrophic effects of a growing heart. The activity of these two enzymes dramatically decreases during adulthood [331]. However, this process is reverted during chronic cardiac hypertrophy when both CDKs 7 and 9 levels are elevated again. Nevertheless, there is a preference to consider CDK9 only as the more important enzyme due to the fact that dominant-negative form of CDK9 was effective in blocking cardiac hypertrophy, whereas dominant-negative form CDK7 was not [332]. Although initially compensatory, enlargement of myocytes can lead to heart failure due to prolonged expression of high levels of CDK9/cyclin T2a.

The immediate response to hypertrophic stimuli is achieved through the activation of the transcriptional mechanism, where P-TEFb complex by phosphorylation of the RNAP-II CTD governs cardiomyocyte specific genes for cell growth and differentiation. Overexpression of CDK9/cyclin T2a complex induces the expression of transcription factors, such as MyoD and enhances myocyte differentiation [333]. In addition, microRNAs were found to have regulatory functions in the progression of cardiac hypertrophy. Especially overexpression analysis of the muscle-specific microRNA-1 (miR-1), a short non-coding RNA involved in muscle differentiation and growth inhibition showed that miR-1 downregulates many growth-related target genes, including CDK9 [334]. Activation of CDK9 in chronic cardiac disorders not only leads to myocyte enlargement, but also suppression of the function of peroxisome proliferator-activated receptor-γ coactivator-1 (PGC-1) [335]. This coactivator is necessary in stimulating mitochondrial function and protein biogenesis, suggesting that suppression of its gene is responsible for the development of heart failure [336]. By developing CDK9 targeted therapies, cardiac hypertrophy or even long term heart diseases could be treated effectively.

## 9. Conclusions

Members of the cyclin-dependent kinase (CDK) family have diverse and unique tissue specific functions. Numerous structural studies have provided detailed mechanistic insights into their distinguishing features and activities. This structural diversity provides useful information for active inhibition which helped to develop new CDK4/6-selective inhibitors to be registered for clinical use in breast cancer treatment. Dysregulation of CDKs and their cyclin partners is observed in a range of tumor types, and some of them have emerged as promising therapeutic targets in cancer. The major challenges in the CDK-targeted drug discovery are selectivity and bad responses, or resistance to treatments. However, the latest advancements in the field provide encouragement that highly selective and potent inhibitors of human cyclin-dependent kinases with favorable pharmacokinetic properties will be identified.

## Data Availability

Not applicable.
